# A rapid evaluation of the preparedness of Ethiopia's disease surveillance system for Mpox outbreak: a cross-sectional study of perspectives from professionals across various levels

**DOI:** 10.1186/s41182-026-00994-8

**Published:** 2026-06-06

**Authors:** Gelan Ayana Zewdie, Henok Gulilat, Ketema Lemma Abdi, Firanol Teshome, Gelane Biru, Hundessa Daba Nemomssa, Tabarak Malik, Million Tesfaye Eshete, Mirkuzie Woldie, Ahmed Zeynudin, Adane Tadesse Gebryu, Netsanet Workneh Gidi, Minyahil Tadesse Boltena, Jude Kong

**Affiliations:** 1 Artificial Intelligence & Mathematical Modeling Lab (AIMMLab), Dalla Lana School of Public Health, University of Toronto, 155 College St, Toronto, ON M5T 3M7 Canada; 2 Africa-Canada Artificial Intelligence & Data Innovation Consortium (ACADIC), Toronto, Canada; 3 Global South Artificial Intelligence for Pandemic and Epidemic Preparedness Network (AI4PEP), Toronto, Canada; 4 School of Biomedical Engineering, Jimma Institute of Technology, Jimma University, 378 Jimma, Ethiopia; 5 Department of Biomedical Sciences, Jimma Institute of Health, Jimma University, 378 Jimma, Ethiopia; 6 Department of Reproductive Health, Faculty of Public Health, Jimma University, 378 Jimma, Ethiopia; 7 Sustainable Health Unit, Faculty of Medicine, University of Oslo, Oslo, Norway; 8 Fenot Associates, Addis Ababa, Ethiopia; 9 School of Medical Laboratory Science, Jimma Institute of Health, Jimma University, 378 Jimma, Ethiopia; 10 Faculty of Electrical and Computer Engineering, Jimma Institute of Technology, Jimma University, 378 Jimma, Ethiopia; 11 Artificial Intelligence and Digital Health Innovation Lab, Armauer Hansen Research Institute, Ministry of Health, 1005 Addis Ababa, Ethiopia

**Keywords:** Mpox, Surveillance, Preparedness, Ethiopia

## Abstract

**Background:**

The potential re-emergence of Mpox poses an increasing public health concern in the Horn of Africa, particularly in Ethiopia. This study examined perceptions of preparedness among surveyed surveillance professionals in Ethiopia regarding the disease surveillance system's ability to detect and respond to a potential Mpox outbreak.

**Methods:**

A descriptive cross-sectional survey design was employed, utilizing a structured 58-item questionnaire that assessed preparedness across five domains: general awareness and understanding, surveillance infrastructure and resources, coordination and communication, preparedness and response, and policy, training, and equity. The survey was distributed to disease surveillance professionals at both federal and regional levels through purposive sampling. The data were analyzed using descriptive statistics, Mann–Whitney *U* tests, Cramér’s V, and content analysis.

**Results:**

Among the 42 surveyed surveillance professionals, 45.3% believed that the surveillance system could effectively respond to an Mpox outbreak, while 54.7% disagreed, reflecting divided perceptions within the sample. Respondents identified several perceived gaps, including limited awareness of Mpox-specific protocols, insufficient training, inadequate diagnostic capacity, and fragmented coordination across sectors. A substantial proportion of respondents reported system-related challenges, with 83.3% perceiving laboratory facilities as inadequate and 78.6% noting the absence of contingency plans. In addition, 57.1% indicated that their organizations lacked staff trained on Mpox, and 59.5% reported no stockpiles of personal protective equipment. Overall, the surveyed professionals expressed mixed perceptions of preparedness, with notable concerns regarding resource allocation, infrastructure, and policy implementation.

**Conclusions:**

The study identifies perceived gaps among the 42 surveyed surveillance professionals regarding Mpox preparedness in Ethiopia, highlighting the need for enhanced training, strengthened infrastructure, improved coordination, and more equitable resource distribution. Addressing these gaps through targeted interventions may help strengthen disease surveillance capacity and improve the ability to detect, respond to, and manage emerging health threats such as Mpox.

**Supplementary Information:**

The online version contains supplementary material available at 10.1186/s41182-026-00994-8.

## Background

Ethiopia has been implementing the Integrated Disease Surveillance and Response (IDSR) strategy since 2000 to strengthen the surveillance of communicable diseases across the country [1]. This strategy helped to integrate disease monitoring into routine healthcare, enabling rapid detection, reporting, and management of public health threats. The IDSR strategy has become a key component of the Public Health Emergency Management (PHEM) system, which is overseen by the Ethiopian Public Health Institute (EPHI), an agency of the Federal Ministry of Health (MoH) [2, 3]. The PHEM system extends from the federal level down to the regional, zonal, and district levels, ensuring that disease surveillance and response are effectively coordinated across all administrative tiers [4]. Regional health bureaus, in collaboration with zonal health departments and district health offices, oversee the implementation of the PHEM system [5]. This decentralized approach ensures that public health monitoring is comprehensive and responsive at the local level. Through this system, more than 20 priority diseases and events have been identified for weekly and immediate reporting, starting from the grassroots level at health posts. These diseases include both endemic threats and emerging diseases [6]. The IDSR–PHEM integration enhanced Ethiopia’s ability to quickly identify outbreaks and coordinate effective responses, ensuring better health security and minimizing the impact of infectious diseases on public health [1, 2, 7].

Despite advancements in surveillance systems over the past two decades because of the IDSR-PHEM integration, the country remains vulnerable to emerging threats due to systemic challenges such as insufficient diagnostic capacity, gaps in healthcare worker training, and dropping funding due to the global dynamics [7–13]. As global health threats like Mpox continue to evolve, the need for a progressive and adaptive disease surveillance system has never been more urgent. In 2022, Mpox cases outside of its endemic regions prompted renewed attention from health authorities worldwide, sparking concerns about its potential spread to countries like Ethiopia, where resources and diagnostic capabilities remain limited [14].

The ability of a country to effectively detect and respond to emerging infectious diseases like Mpox relies on the robustness of its disease surveillance system. Preparedness, in this context, is defined as the capacity of health authorities to anticipate, detect, and respond to public health threats in a timely and efficient manner. The global experience with pandemics, particularly the COVID-19 pandemic, has emphasized the importance of rapid, coordinated responses to emerging diseases. During COVID-19, many countries, including Ethiopia, adapted their healthcare systems to meet the demands of the disease [15]. However, the lessons learned from COVID-19 regarding the need for better preparedness, clear communication, and robust systems integration have yet to be fully applied to emerging diseases like Mpox [16, 17]. The lack of a specific surveillance framework for Mpox and the underutilization of innovative tools for surveillance and reporting further hinder Ethiopia's preparedness [18].

This research seeks to evaluate the readiness and preparedness of Ethiopia's disease surveillance system, specifically in response to a potential Mpox outbreak. By identifying strengths and weaknesses in Ethiopia’s current public health infrastructure, the study aims to provide actionable recommendations to improve the nation’s ability to respond effectively to Mpox and other future emerging infectious disease threats.

### Aims

This study investigates the knowledge, perceptions, and readiness of Ethiopia’s disease surveillance system to respond to a potential Mpox outbreak. Specifically, the study aims to explore:

- The readiness of Ethiopia’s disease surveillance system, including health institutions at federal, regional, and local levels, to detect and respond to a potential Mpox outbreak.
- The perceived effectiveness of current surveillance systems, including infrastructure, resources, and personnel capacity, to handle an Mpox outbreak.
- The knowledge and awareness of public health professionals regarding Mpox, including its symptoms, transmission routes, and the capacity of the health system to respond effectively.
- The role of the existing health policies, training programs, and community involvement in preparing for an Mpox outbreak, with particular emphasis on gender-sensitive and equity-focused approaches.
- The challenges and gaps in Ethiopia’s disease surveillance system, including issues related to diagnostic capacity, communication, and coordination across health sectors, and the implications for outbreak response.
- Recommendations for enhancing Ethiopia's disease surveillance and preparedness efforts, based on findings regarding system strengths, weaknesses, and areas for improvement.

## Literature review

Several studies have assessed the readiness of Ethiopia’s national disease surveillance system to detect and respond to emerging diseases, highlighting both notable progress and persistent challenges [2, 7, 19–22]. A prominent evaluation conducted in Dangila district (Amhara region) used the U.S. Centers for Disease Control and Prevention (CDC)’s surveillance system evaluation framework to assess core attributes such as simplicity, flexibility, and usefulness [23]. The study found high reporting completeness (100%) and timeliness (94.6%), indicating a well-functioning reporting mechanism. However, it also revealed critical weaknesses in data analysis, interpretation, and feedback dissemination, functions that are essential for early warning and rapid response, particularly in the context of emerging infectious diseases.

Another evaluation focused on the Maternal Death Surveillance and Response (MDSR) system, a component of Ethiopia’s broader surveillance infrastructure [24]. Although the system demonstrated moderate readiness in terms of detection and notification, it fell short in translating data into actionable insights. This gap between data collection and decision-making highlights a broader issue across the surveillance system: the lack of consistent use of surveillance data to guide public health responses to emerging threats. Studies assessing surveillance readiness for vaccine safety monitoring, particularly the Adverse Events Following Immunization (AEFI) system, have also raised concerns. A 2021 assessment in Addis Ababa revealed that only one of three hospitals met basic readiness criteria, with major gaps in trained personnel, access to updated guidelines, and integration of digital reporting tools [25]. These limitations are particularly concerning given the need for rapid deployment and monitoring of new vaccines during emerging outbreaks. One study [26] specifically assessed the confidence levels of healthcare workers (HCWs) in diagnosing and managing Mpox in hospitals across Ethiopia’s Amhara region, finding that only 31.5% reported high confidence, while 41.8% had low confidence. Factors significantly associated with higher confidence included being a physician, aged 30–35, having received epidemic management training, holding a positive attitude, and regularly accessing reliable online health resources. The study concludes that targeted training and continuous professional development are urgently needed to enhance HCWs' preparedness for managing emerging diseases like Mpox.

Although most evaluations focus on specific diseases or components, their findings reflect systemic challenges affecting Ethiopia’s preparedness for emerging diseases more broadly. For instance, research on malaria elimination efforts, although centered on an endemic disease, has exposed deficiencies in diagnostic capacity, supply chain reliability, and trained human resources. These weaknesses are equally relevant for the detection and confirmation of novel pathogens, underscoring the importance of robust, cross-cutting surveillance infrastructure. The COVID-19 pandemic has served as a real-world stress test of the system's capacity. Although Ethiopia demonstrated improvements in emergency coordination and the activation of national and regional Public Health Emergency Operations Centers (PHEOCs), studies noted gaps in real-time data sharing, inter-sectoral coordination, and the inclusion of community-level data in national response planning [27, 28]. Despite national adoption of frameworks, such as the IDSR and the PHEM strategy, the implementation of these systems at subnational levels remains inconsistent [1].

In general, the literature evaluating Ethiopia’s disease surveillance system reflects a dual reality: a strong policy and structural foundation exists, but operational readiness, particularly for emerging diseases, remains uneven. To enhance preparedness, studies consistently recommend strengthening laboratory capacity, improving training and retention of health personnel, and reinforcing analytical and feedback capabilities at the district and regional levels. Addressing these gaps is critical for transforming the surveillance system into an agile and responsive tool for managing both known and emerging public health threats.

## Methodology

**Fig. 1:**
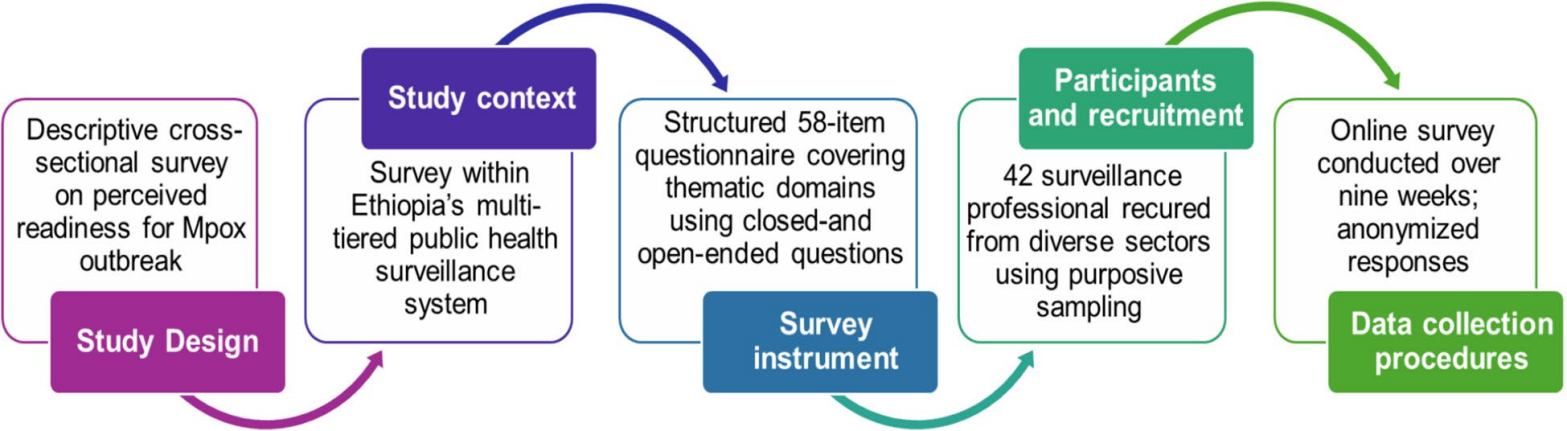
Study methodology flowchart

The study employed a descriptive cross-sectional survey design to evaluate Ethiopia's preparedness for a potential Mpox outbreak (Fig. 1), concentrating on critical domains including awareness, infrastructure, coordination, and response. A purposive sampling approach was used to select 42 surveillance professionals, and data were gathered through a secure online platform over a nine-week period, ensuring both anonymity and voluntary participation.

### Study design

A descriptive cross-sectional survey design was employed to collect both quantitative and qualitative data on the perceived preparedness of Ethiopia’s disease surveillance system to respond to a potential Mpox outbreak. The design allowed the timely collection of insights from a diverse cohort of surveillance professionals, capturing a broad perspective on the system’s strengths and limitations. This study is reported in accordance with the STROBE (Strengthening the Reporting of Observational Studies in Epidemiology) guidelines (supplementary file 1).

### Study context

The study was situated within Ethiopia’s multi-tiered public health surveillance system, which encompasses federal, regional, zonal, woreda (district), and health facility levels. This system is coordinated by the Federal Ministry of Health and the Ethiopian Public Health Institute, in collaboration with regional health bureaus, academic institutions, nongovernmental organizations (NGOs), and private sector stakeholders. At the time of data collection, no Mpox cases had been confirmed in Ethiopia; however, confirmed cases in neighboring countries and global alerts underscored the urgency of assessing national preparedness. Ethiopia’s first confirmed Mpox case was reported on May 25, 2025. Consequently, most responses reflect perceptions formed prior to in-country case confirmation, while data analysis was conducted after this initial case had been identified.

### Survey instrument

A structured 58-item questionnaire (Supplementary file 2) was developed based on internationally recognized public health emergency preparedness frameworks [29–32] and adapted to the Ethiopian context [23, 26]. The questionnaire covered five key thematic domains, including general awareness and understanding, surveillance infrastructure and resources, coordination and communication, preparedness and response, and policy, training, and equity. Each domain included closed-ended items using a combination of Likert-type scales (three-, four-, and five-point), binary responses (e.g., yes/no), and open-ended questions to capture contextual insights. The questionnaire was reviewed by two public health specialists and piloted among a small group of surveillance professionals, leading to minor revisions to improve clarity and comprehensibility.

#### General awareness and understanding domain

This domain consisted of 10 items designed to assess respondents’ general awareness and understanding of Mpox. Participants were asked about their familiarity with Mpox symptoms, perceptions of its public health threat in Ethiopia, and understanding of transmission routes (e.g., animal contact, human-to-human contact, contaminated objects). The questions also explored respondents’ primary sources of information on emerging diseases, perceived preparedness of the national health surveillance system, and frequency of policy updates. In addition, the domain evaluated training experiences related to Mpox or similar diseases, perceptions of the health infrastructure's capacity for case detection, and the reliability of different surveillance data sources. Respondents were also invited to identify perceived gaps in Ethiopia’s ability to detect and respond to Mpox outbreaks.

#### Surveillance infrastructure and resources domain

This domain comprised 10 items assessing respondents’ perspectives on the infrastructure and resources available for Mpox surveillance. Questions explored the presence of dedicated staff for infectious disease surveillance, the adequacy of human resources, and the availability and accessibility of laboratory facilities, particularly in rural areas. Respondents were also asked about the use of technological tools for data collection and reporting, the reporting capacity of healthcare facilities, and which resources (e.g., staff, equipment, training, or funding) were most lacking. In addition, the domain evaluated the existence of reporting systems such as DHIS2, the frequency of routine surveillance meetings, and solicited recommendations for improving Mpox diagnostic capacity.

#### Coordination and communication domain

This domain comprised 10 items that assessed respondents’ perceptions of coordination and communication related to Mpox surveillance and response. Questions explored the effectiveness of coordination among public health organizations, clarity in reporting hierarchies, and the efficiency of communication between federal and regional offices. The involvement of community health workers, commonly used communication tools (e.g., mobile apps, emails, phone calls, and paper reports), and challenges in data sharing were also examined. Respondents were asked about the timeliness of updates from the Ministry of Health, the presence of public awareness campaigns, and the engagement level of nongovernmental organizations. Suggestions for improving coordination and communication among stakeholders were also solicited.

#### Preparedness and response domain

This domain comprised 10 items aimed at assessing respondents’ perceptions of their organization's preparedness and response capacity for a potential Mpox outbreak. Questions explored the existence of contingency plans, the availability of personal protective equipment (PPE) stockpiles, the frequency of simulation exercises, and the adequacy of outbreak funding. Respondents were also asked to evaluate their organization’s capability to respond to Mpox, the number of staff trained in outbreak protocols, and the preparedness of isolation and treatment facilities. Additional items examined access to public education resources and barriers to effective response, while an open-ended question invited suggestions on priority actions for improving Mpox preparedness.

#### Policy, training, and equity domain

This domain comprised 10 items designed to assess the state of policy, training, and equity in relation to Mpox preparedness and surveillance. Respondents were asked about the existence and adequacy of policies for managing zoonotic disease outbreaks, the availability of training opportunities (including gender-sensitive approaches), and the consideration of gender-disaggregated data in surveillance systems. The items also explored the inclusivity of marginalized groups, the use of local languages in outreach, and efforts to involve underserved communities. Additional questions sought suggestions for policy adjustments and resource needs to ensure equity in Mpox preparedness efforts.

### Participants and recruitment

Participants were professionals involved in disease surveillance and outbreak preparedness across diverse sectors in Ethiopia, including public health institutions at all administrative levels, academic and research institutions, NGOs, and private health facilities. This was an exploratory descriptive study; therefore, no formal sample size calculation was performed. The sample size was based on the number of eligible surveillance professionals available and willing to participate during the study period.

A purposive sampling strategy was employed to recruit individuals with direct relevance, expertise, or experience in Ethiopia’s disease surveillance system, particularly in the context of Mpox preparedness. Because the study aimed to assess system-level readiness, it was essential to include participants actively engaged in surveillance operations, public health policy, outbreak response, or health facility focal roles. These roles are not randomly distributed in the population; therefore, random sampling would have been inefficient and potentially uninformative. Purposive sampling enabled the intentional inclusion of key informants whose perspectives are critical for identifying system-level strengths and gaps, thereby enhancing the validity and contextual relevance of the findings.

Invitations were sent to 62 professionals across regional and federal health sectors, of whom 42 completed the survey, yielding a response rate of 67.74%. Invitations were distributed via institutional email lists, formal letters, and professional networks, leveraging existing partnerships to ensure access to relevant respondents while facilitating trust, legitimacy, and cooperation. This approach also supported representation across administrative levels and sectors (e.g., federal, regional, and facility-based actors).

### Data collection procedures

Data collection was conducted over a nine-week period, from March 13 to May 26, 2025, using a secure, web-based survey platform accessible via internet-enabled devices. The questionnaire was self-administered and presented in English. To maximize response rates, periodic reminders were sent to participants throughout the data collection period. All responses were collected anonymously, and the dataset was securely stored with access restricted exclusively to the research team.

Participation was voluntary, and informed consent was obtained from all participants prior to survey access. The study received ethical approval from Jimma University Institute of Health Institutional Review Board (Ref. No: JUIH/IRB/0460/25).

**Fig. 2:**
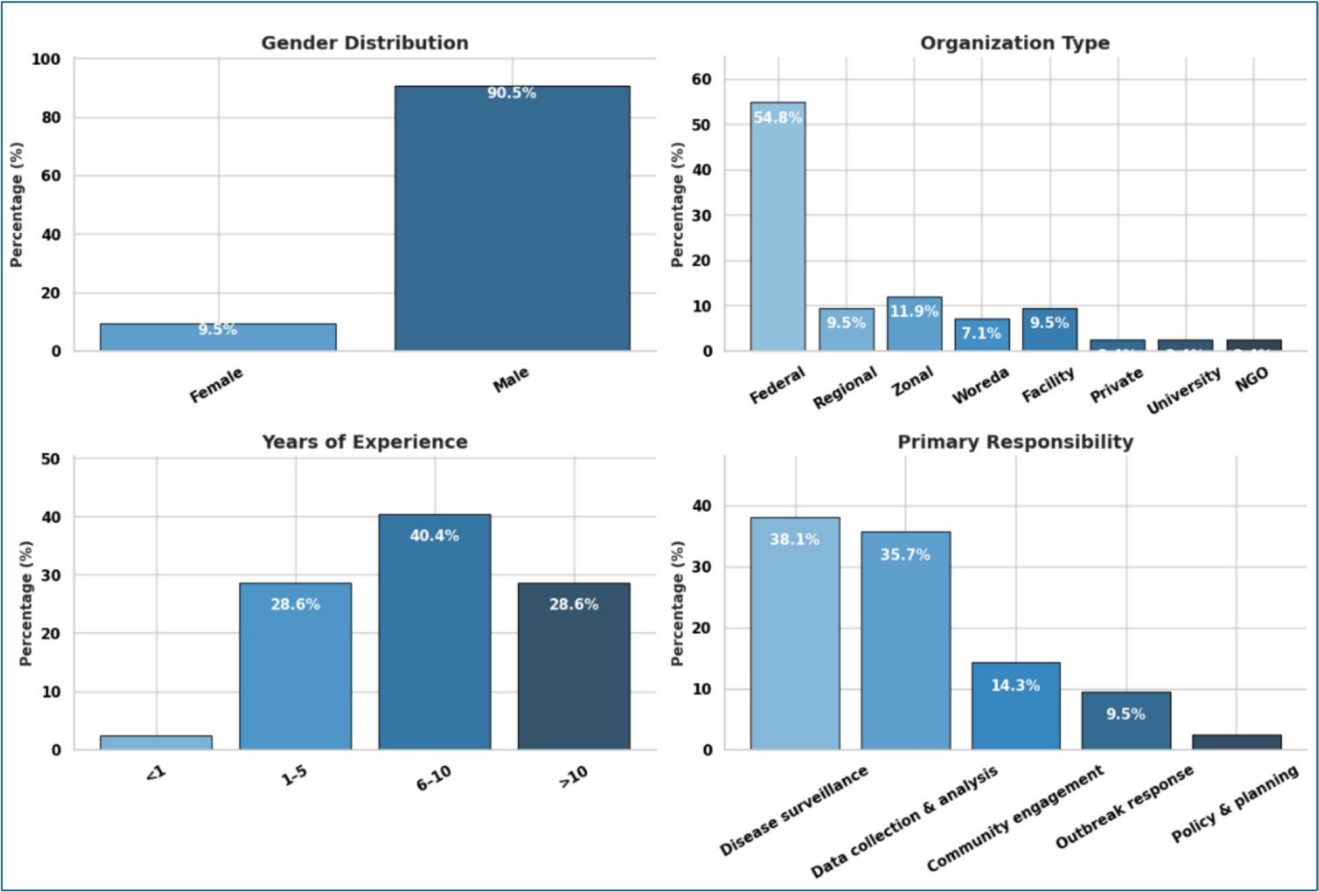
Demography of respondents

As shown in Fig. 2, most respondents were male (90.5%) and primarily affiliated with federal institutions (54.8%), with others representing regional, zonal, woreda, facility, private, university, and NGO sectors. Most had between 6–10 years (40.4%) or 1–5 years (28.6%) of experience. Their primary responsibilities included disease surveillance (38.1%), data collection and analysis (35.7%), community engagement (14.3%), outbreak response (9.5%), and policy and planning (2.4%). The distribution of participants across multiple levels and institutions enabled a comprehensive understanding of the surveillance system.

### Data analysis

Quantitative data were cleaned and analyzed using SPSS version 29. Descriptive statistics, including frequencies, percentages, means, and standard deviations, were calculated to examine overall trends and subgroup variations across the five thematic areas. Likert-scale responses were consolidated into categories (e.g., “agree/strongly agree” and “disagree/strongly disagree”) for ease of interpretation; “undecided” responses were analyzed separately, and “not applicable” responses were excluded from analysis. Because the questionnaire included items with heterogeneous response formats (binary, 3-point, and 4-point ordinal scales), item responses were normalized before aggregation to ensure comparability across different scoring ranges. A scale-specific min–max normalization (linear rescaling to a 0–1 range) was applied using the formula: Xnorm​ = Xmax​ − Xmin ​/ X − Xmin​​, where *X* represents the original item score, *X*ₘᵢₙ is the minimum possible score, and *X*ₘₐₓ is the maximum possible score for the respective item. Accordingly, 4-point scale items were transformed using (score − 1)/3, 3-point scale items using (score − 1)/2, and binary items using score/1, yielding normalized values ranging from 0 to 1. In SPSS, normalized variables (Q1_norm–Q40_norm) were generated and used in all subsequent analyses. Domain scores were computed by aggregating normalized item responses within each thematic domain. Specifically, the mean score of normalized items within each domain was calculated for each respondent. For example, the knowledge domain score was computed as the average of items Q1–Q4, while the surveillance domain was calculated as the mean of items Q5–Q17. Similarly, coordination (Q18–Q24), preparedness (Q25–Q34), and policy (Q35–Q40) domain scores were derived by averaging their respective normalized items. This approach ensured that each item contributed equally to the overall domain score while accounting for differences in original response scales.

To assess differences between trained and untrained respondents, we first applied the Kolmogorov–Smirnov test to evaluate whether score distributions were normal. Several items demonstrated significant non-normality, indicating that parametric tests such as t-tests were unsuitable. Given the ordinal nature of the Likert-scale data, small subgroup sizes, and skewed distributions, the Mann–Whitney U test was selected as an appropriate nonparametric alternative. This test compares the ranks of responses between independent groups without assuming normality or equal variances, ensuring robust analysis. It was used to determine whether training exposure significantly influenced perceptions of surveillance infrastructure and overall preparedness. Cramér’s V was then used to assess the strength of association between categorical variables, such as the availability of trained staff and the readiness of isolation facilities. This method was selected for its ability to measure the strength of relationships between nominal variables, helping to identify critical interdependencies within the surveillance system. Strong associations highlighted key areas, such as the correlation between staff training and the preparedness of treatment facilities. To further visualize these relationships, a heatmap was created to represent the statistical significance of associations between key variables. The heatmap provided a clear, intuitive view of which factors, such as laboratory access and funding perceptions, were most strongly linked, helping to prioritize areas for intervention. A boxplot analysis was performed to compare responses based on the years of experience of participants. This analysis helped identify whether respondents with more public health experience perceived Ethiopia’s preparedness differently from those with less experience. The Mann–Whitney U test was applied to these groups as well to assess whether these differences were statistically significant.

Qualitative data from open-ended responses were analyzed using content analysis. The analysis followed these steps:

- Decontextualization: Extracting meaningful statements and assigning thematic codes.
- Recontextualization: Verifying coding accuracy by reviewing statements in context.
- Categorization: Grouping codes into themes and sub-themes.
- Compilation: Synthesizing findings into a narrative form, supported by anonymized participant quotations.

To ensure the reliability of the qualitative coding, two researchers independently coded all open-ended responses, and inter-coder agreement was assessed using Cohen’s kappa for each item. Six items demonstrated substantial agreement (κ > 0.70), while the remaining items showed lower levels of agreement (κ < 0.70). For items with lower agreement, discrepancies between coders were systematically reviewed and resolved through discussion until consensus was reached. These results indicate an overall acceptable level of agreement, with additional consensus procedures applied to ensure consistency in coding. All completed questionnaires were checked for completeness, and no significant missing data were identified; therefore, all responses were included in the final analysis.

## Results

The study reveals significant gaps in Ethiopia’s preparedness for a potential Mpox outbreak. While 88% of respondents were aware of Mpox symptoms, transmission knowledge was inconsistent, and 83.3% considered it a major threat. The key gaps include inadequate laboratory facilities (83.3%), limited rural testing access (66.6%), and fragmented communication and coordination. Perceptions of response readiness were mixed, with 45.3% viewing the system as capable or highly capable of responding to an Mpox outbreak, while the remainder perceived only partial readiness or inadequate response capacity. In addition, 78.6% reported a lack of contingency plans. Training and PPE shortages were also highlighted, with 57.1% lacking trained staff. Table 1 summarizes the results of this study.

**Table 1:** Ethiopian readiness for the Mpox outbreak results summary

Domain	Key findings	Percentage of respondents (*n* = 42)	Additional details
General awareness and understanding	88% aware of Mpox symptoms, but knowledge is inconsistent	6 (14.3%) very familiar 5 (12%) not at all familiar 16 (38%), familiar 15 (35.7%) somehow familiar	Perceived as a foreign disease, affecting prioritization in Ethiopia
	83.3% view Mpox as a significant threat	35 (83.3%) respondents believe Mpox is a significant threat	Indicates perceived urgency for preparedness
	Transmission knowledge: 50% human-to-human, 40.47% animal contact, 7.1% fomites	21 (50%) human-to-human, 17 (40.47%) animal contact, 3 (7.1%) contaminated objects. Only 1 (2.38%) do not know	Transmission knowledge moderate, 2.4% unsure
	Most used information sources: World Health Organization (WHO), CDC, social media, and local institutions	70.2% rely on formal sources (WHO, CDC); 29.8% use informal sources	Misinformation through social media remains a concern
	Mixed perceptions of Ethiopia’s surveillance system: 40.47% rated it as "Good," 23.8% as "Average," 21.4% as "Poor," and 14.3% as "Excellent."	17 (40.47%) rated "Good", 10 (23.8%) "Average", 9 (21.4%) "Poor", 6 (14.3%) "Excellent."	Mixed confidence in the effectiveness of the surveillance system
Surveillance infrastructure and resources	83.3% found current lab facilities inadequate; 66.6% reported a lack of rural testing access	35 (83.3%) find lab facilities inadequate; 26 (66.6%) report lack of rural access	Gaps in diagnostic capabilities, particularly in rural areas
	57.1% have no Mpox-specific reporting mechanisms	24 (57.1%) respondents report no reporting mechanisms	Fragmented surveillance efforts
	47.6% rated human resources for Mpox as inadequate	20 (47.6%) rated human resources as inadequate	Only 26.2% rated resources as adequate
	57% have access to DHIS2 and Kobo toolbox, but 33% say they are underutilized for Mpox surveillance	24 (57%) have access to tools, but 14 (33%) report underutilization	Disparities in tool usage and accessibility
	64.28% cited lack of funding as a key resource gap	11 (26.19%) identified a lack of training as a major gap, 27 (64.28%) funding, 3 (7.14%) equipment, and 1 (2.38%) staff gap	Funding is the most commonly reported missing resource
Coordination and communication	54.8% rated communication between federal and regional authorities as moderately effective	20 (47.6%) rated communication moderately effective, 10 (23.8%) effective, 10 (23.8%) somewhat effective, 2 (4.76%) ineffective	Minimal fragmented communication between levels of government
	59.5% reported a clear reporting chain	25 (59.5%) reported a clear reporting chain	Gaps in coordination and unclear roles
	50% said community health workers (CHWs) were involved in surveillance activities	21 (50%) involved CHWs in surveillance activities	A significant portion is excluded from surveillance efforts
	45.2% used instant messaging apps for communication, 33.3% used phone calls	19 (45.2%) used messaging apps, 14 (33.3%) used phone calls, 6 (14.28%) used paper reports, and 3 (7.14%) used email	Use of digital communication tools, with low reliance on traditional methods
	30.96% reported public awareness campaigns; 69.04% indicated no such campaigns took place	13 (30.96%) reported campaigns, 29 (69.04%) reported no campaigns	Gaps in public outreach and education efforts
Preparedness and response	16.7% believed the system could effectively (highly capable) respond to an Mpox outbreak	7 (16.7%) highly capable; 12 (28.6%) capable; 11 (26.1%) somewhat capable; 12 (28.6%) not capable	More than half of the respondents believe that the system is adequately prepared
	78.6% noted lack of contingency plans	33 (78.6%) reported no contingency plans	Lack of contingency plans is a critical gap
	57.1% of organizations lacked trained staff; 61.9% lacked PPE stockpiles	24 (57.1%) lacked trained staff, and 26(61.9%) lacked PPE	Training and protective equipment shortages
	52. 38% had never conducted simulation exercises, 26.19% had done so rarely	22 (52.38%) never conducted simulations, 11 (26.19%) rarely, 5 (11.9%) annually, and 4 (9.52%) biannually conducted simulation	Insufficient preparedness exercises
	80.95% rated funding as inadequate	34 (80.95%) rated funding as inadequate, 5 (11.9%) as somewhat adequate, and 3 (7.14%) as adequate	Funding gaps severely limit response capacity
Policy, training, and equity	71.4% reported existing zoonotic disease policies; 28.6% lacked such policies	30 (71.4%) reported zoonotic policies, 12 (28.6%) reported no policies	Policies are in place, but many gaps in coverage for Mpox
	52.4% rated the Mpox-specific policy as somewhat adequate	22 (52.4%) rated the policy as somewhat adequate, 9 (21.42%) as well, 5 (11.9%) as very well, and 6 (14.28%) as not at all	The majority views the Mpox policy as insufficient
	21.42% received gender-sensitive training; 30.95% collect gender-disaggregated data	9 (21.42%) received gender-sensitive training, 13 (30.95%) collect gender-disaggregated data	Lack of inclusivity and gender-sensitive approaches in training
	59.5% used local languages in outreach; 40.5% did not	25 (59.5%) used local languages, 17 (40.5%) did not	Gaps in outreach to local communities
	Recommendations: Integration of Mpox into national systems, decentralization of labs, increased funding, gender and equity integration, One Health approach		Strong call for integrating Mpox into existing systems and increasing equity

### General awareness and understanding

Most respondents were aware of Mpox; however, their knowledge regarding its transmission, symptoms, and epidemiology was inconsistent. The disease was also frequently perceived as a foreign health problem, which appeared to influence its prioritization in Ethiopia. Although 88% of respondents reported awareness of Mpox symptoms, only 14.3% considered themselves very familiar with the disease, while 12% reported no familiarity at all. Nevertheless, 83.3% perceived Mpox as a significant public health threat, underscoring the urgent need for strengthened preparedness efforts.

Transmission knowledge among respondents was moderately strong, with 50% identifying human-to-human contact as a mode of transmission, 40.47% recognizing animal contact, and 7.1% mentioning fomites; only 2.38% expressed uncertainty. Respondents reported using a range of information sources, with the World Health Organization (WHO) and the CDC being the most frequently cited, followed by social media platforms and local institutions. This reliance on both formal and informal channels underscores the importance of strengthening critical appraisal skills, particularly given the potential for misinformation through unverified sources.

Perceptions of surveyed personnel in the surveillance system were mixed, 40.47% rated it as "Good," 23.8% as "Average," 21.4% as "Poor," and 14.3% as "Excellent." Policy updates frequency was inconsistent, with 38.1% rarely receiving updates, while 33.3% received them weekly. Only 23.8% of respondents had Mpox-related training, indicating significant capacity gaps.

Of those trained, content varied widely; some received formal instruction on surveillance and laboratory methods, while others had only community-level orientations or training unrelated to Mpox. Respondents cited major gaps in lab decentralization, training, public awareness, and outdated manual reporting systems. Only four respondents saw no significant gaps, reinforcing broad concerns.

### Surveillance infrastructure and resources

Many respondents pointed to a critical shortage of Mpox diagnostics, particularly in regional areas, and emphasized that even federal laboratories lacked access to PCR testing. Surveillance efforts for Mpox remain fragmented, as the disease is not included in Ethiopia’s routine surveillance systems. The only exceptions were limited monitoring activities conducted at airports and in the Ethio-Kenya border city of Moyale, where the country’s first Mpox case was identified. Only 2 (4.8%) respondents indicated that more than 75% of facilities in their area could report Mpox cases. Most reported less than 50% coverage.

An overwhelming 83.3% found current lab facilities inadequate, and 66.6% reported a lack of rural testing access. Nearly half (47.6%) rated human resources for Mpox as inadequate. Only 26.2% considered them adequate. On tech tools, 57% reported access to platforms like DHIS2 and Kobo toolbox, but 33% said tools were unavailable or underutilized for Mpox.

Reporting mechanisms for Mpox were not in place for 57.1% of respondents. Surveillance meeting frequency varied, with 52.4% held weekly and others biweekly, monthly, or quarterly. Training was the top lacking resource (54.8%), followed by funding (38.1%) and equipment (7.1%).

To improve diagnostics, 67% prioritized training; 52% called for improved infrastructure and decentralization; 33% emphasized surveillance enhancement. Funding, logistics, and governance also emerged as key needs.

### Coordination and communication

Coordination was seen as fragmented. Communication between federal and regional authorities was rated moderately effective by 47.6% of respondents. Only 23.8% rated it fully effective. Surveillance activity coordination was viewed as "well" by 42.9% and "very well" by 26.2%, though 31% rated it somewhat or not at all coordinated.

Only 59.5% of respondents reported having a clear reporting chain, highlighting a fragmented communication structure. This fragmentation extended to other areas, with 52.4% noting that community health workers (CHWs) were involved in surveillance activities, while 47.6% reported their exclusion. In terms of communication tools, instant messaging apps were the most commonly used, cited by 52.4% of respondents, followed by phone calls at 33.3%. Traditional methods such as paper-based communication and email were less frequently used, reported by 11.9% and 7.1% of respondents, respectively.

The timeliness of outbreak information varied considerably among respondents. While 28.6% reported receiving updates immediately and 23.8% within 24 h, a significant 47.6% experienced delays of more than three days. Public awareness campaigns were reported by only 35.7% of respondents, whereas 64.3% indicated that no such campaigns had taken place. Engagement from NGOs was generally perceived as low, with 52.4% of participants rating it as limited and just 4.8% describing it as highly involved.

Respondents suggested establishing national task forces, multisectoral collaboration, clear standard operating procedures (SOPs), digital platforms, and regular simulations. Emphasis was placed on trust, feedback, and inclusive coordination models.

### Preparedness and response

Preparedness perceptions were mixed. Overall, 45.3% of respondents considered their organizations capable of responding to an Mpox outbreak, comprising 16.7% who rated their organizations as highly capable and 28.6% who rated them as capable. Meanwhile, 26.1% perceived their organizations as somewhat capable, and 28.6% considered them not capable. A lack of contingency plans was reported by 78.6% of respondents.

Training gaps were stark, with 57.1 percent of organizations lacking trained staff. In addition, 61.9 percent of facilities reported an absence of PPE stockpiles. Simulation exercises were also infrequent, as 52.38 percent of respondents indicated they had never conducted them, while 26.19 percent had done so only rarely. Overall, preparedness was found to be severely constrained by deficits in funding, training, essential equipment, and effective communication.

Only 31% of respondents had access to public education materials. On funding adequacy, 78.6% rated it as inadequate. Isolation and treatment facilities were unprepared according to 73.8%. Collectively, these findings reveal serious shortcomings in preparedness and response capacity, highlighting critical gaps in training, simulation exercises, protective equipment availability, and coordinated emergency planning.

Key steps prioritized by respondents included training and capacity building, which was mentioned by 15 participants, followed by strengthening surveillance with 12 mentions. Improving case management was highlighted by 10 respondents, while risk communication was noted by 9. Resource mobilization received 8 mentions, multisectoral coordination was emphasized by 7, and the development of preparedness plans was cited by 6. Finally, enhancing diagnostic capacity was identified as a priority by 5 respondents.

### Policy, training, and equity

Most respondents (71.4%) reported existing zoonotic disease policies, but 28.6% lacked such policies. On Mpox-specific policy adequacy, 52.4% said "somewhat," 21.42% "well," 11.9% "very well," and 14.28% "not at all." Training opportunities were fragmented; some cited WHO or Africa CDC training, but many reported no access.

Only a small proportion of respondents, 21.42%, had received gender-sensitive training, and just 30.95% reported that their surveillance systems collect gender-disaggregated data. Inclusivity remains a significant challenge, with nearly half of the respondents describing the surveillance system as less inclusive and over one-fifth stating that it is not inclusive at all.

While 59.5% reported use of local languages in outreach, 40.5% did not. Respondents noted engagement strategies like community health worker mobilization, brochures, and multilingual education. However, others reported "no action" or a lack of MOH inclusion.

Respondents called for integration of Mpox into national HMIS and PHEM systems, decentralization of labs, increased funding, community engagement, gender and equity integration, and cross-border collaboration. A One Health approach was frequently recommended.

Equity-related needs included rural diagnostics, frontline training, public health education in local languages, and tools for disaggregated data. Respondents highlighted policy gaps, limited community trust, and a lack of political will. Equitable Mpox preparedness demands structural reforms, community inclusion, and sustained investment.

### Further analysis

Further quantitative framing helps identify which elements of the surveillance and response ecosystem are most interdependent and where policy or programmatic adjustments may yield the greatest impact.

The Cramér’s V analysis revealed three notable associations among key variables. The strongest relationship was found between the number of trained staff within an organization and the readiness of isolation and treatment facilities (Cramér’s *V* = 0.73), indicating that institutions with more trained personnel were substantially more likely to have prepared treatment settings. The second highest association (Cramér’s *V* = 0.69) was between perceptions of human resource adequacy and the presence of contingency plans, showing that workforce strength aligns closely with proactive planning. Lastly, accessibility of laboratory services was moderately associated with perceptions of funding adequacy (Cramér’s *V* = 0.55), suggesting that perceptions of underfunding may be rooted in the reality of limited diagnostic infrastructure. All three associations point to structural interdependencies, highlighting that training, funding, and resource planning are not isolated domains but co-vary meaningfully across Ethiopia’s Mpox preparedness landscape.

**Fig. 3:**
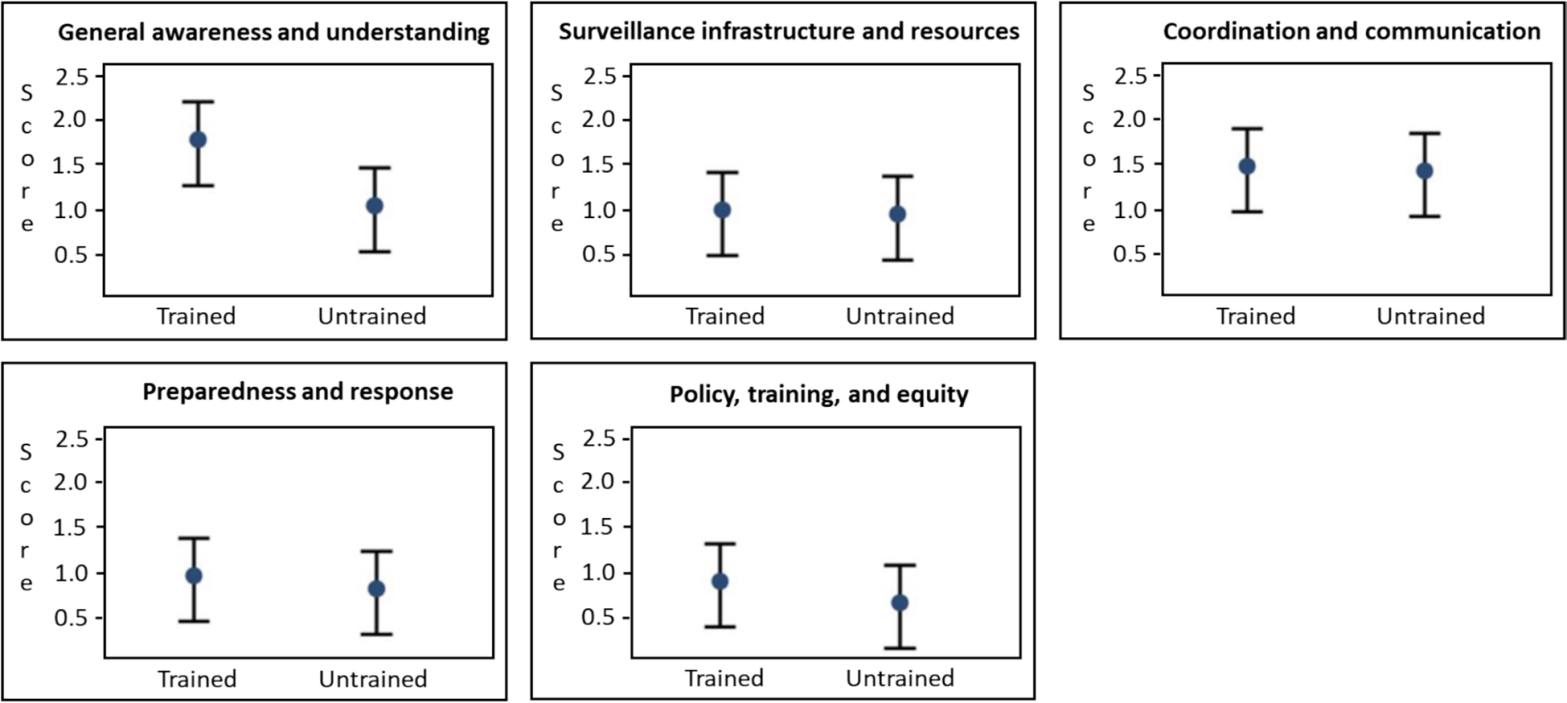
Median score comparison visualization of the five themes by Mpox training status

To statistically assess whether differences across groups were meaningful, we applied the Mann–Whitney U test, a nonparametric method appropriate for comparing two independent groups without assuming normal distribution. For each theme, respondents’ scores were computed by averaging responses to related items, then ranked and compared between trained and untrained groups. Across all thematic domains, no statistically significant differences were observed (all *p*-values > 0.05). However, given the relatively small sample size (trained *n* = 10 vs. untrained *n* = 32), this analysis is underpowered and therefore only capable of detecting very large effect sizes (approximately Cohen’s d ≈1.0) with adequate power (80%). As such, the absence of statistically significant differences should be interpreted with caution. Figure 3 provides a comparative analysis of respondents’ scores across five key thematic areas: General Awareness and Understanding, Surveillance Infrastructure and Resources, Coordination and Communication, Preparedness and Response, and Policy, Training, and Equity, distinguished by whether participants had received Mpox-related training. For General Awareness, those with training showed a slightly higher median score (1.79) than untrained respondents (1.29), suggesting a modest trend toward improved familiarity with Mpox, although the difference was not statistically significant (*p* = 0.116). In Surveillance Infrastructure, trained and untrained respondents had nearly identical medians (both approximately 1.0), with a *p*-value of 0.949, reflecting no meaningful difference. Similar patterns were observed in Coordination and Communication (medians: trained 1.52 vs. untrained 1.50, *p* = 0.899), Preparedness and Response (trained 1.0 vs. untrained 0.92, *p* = 0.727), and Policy, Training, and Equity (trained 0.8 vs. untrained 0.6, *p* = 0.620). Taken together, these findings indicate that this study did not have sufficient statistical power to detect an association between individual training exposure and perceived preparedness. Larger, adequately powered studies are needed to more definitively evaluate the effect of training on preparedness outcomes. The same analysis was performed to get insights regarding the differences in how public health professionals with varying levels of experience (High experience (professionals with more than five years of experience) vs. Low experience (professionals with less than five years of experience)) perceive Mpox preparedness across five thematic domains. Across all themes, Mann–Whitney *U* tests returned *p* values above the 0.05 threshold, indicating no statistically significant differences between the two experience groups. This uniformity in perceptions across experience levels suggests that barriers to Mpox readiness in Ethiopia are more systemic than experiential, rooted in institutional constraints and policy gaps rather than differences in individual background or tenure. Moreover, an analysis comparing respondents based on whether they perceive Mpox as a significant public health threat (those who recognized Mpox as a significant threat vs. those who didn’t) in Ethiopia was performed to see patterns across the five preparedness themes. Overall, while no theme crossed the significance threshold (*p* < 0.05), the consistent directionality of higher scores among those who acknowledged Mpox as a threat suggests that risk perception may influence how system strengths and gaps are evaluated, even if the current sample size limits statistical power. These findings highlight the importance of fostering accurate risk perception to align institutional awareness with operational readiness.

## Discussion

The findings from this study, based on the purposive sampling of surveillance professionals in Ethiopia, present a complex picture of Mpox preparedness within their respective work settings. While a substantial majority of respondents demonstrated general awareness of Mpox and recognized it as a significant public health threat, this awareness was not consistently matched by operational readiness. Only a minority reported deep familiarity with Mpox symptoms or having received relevant training. The observed knowledge–action gap highlights the limitations of awareness alone in ensuring preparedness for outbreak response. Without structured training and continuous capacity-building initiatives, even well-intentioned health professionals may face challenges in responding effectively when cases arise. Moreover, the reliance on global institutions such as the WHO and CDC for information, alongside informal channels like social media, underscores the need for locally adapted, standardized, and regularly updated training and communication materials.

Surveillance system infrastructure emerged as a critical point of concern. Diagnostic capacity, particularly in rural and regional areas, remains severely limited, with only 2 (4.8%) of respondents indicating that a majority of local facilities can report Mpox cases. The absence of Mpox from routine IDSR tools and insufficient use of digital reporting platforms compound these limitations, leading to underreporting and delayed response. In addition, the underutilization of tools like DHIS2 and Kobo toolbox, coupled with irregular surveillance meetings, points to a lack of system-wide standardization and coordination. The findings clearly indicate that greater investment in diagnostic decentralization, reporting tools, and routine surveillance processes is needed, as respondents’ views suggest that the country may remain vulnerable to missed cases and delayed outbreak detection and response.

Coordination and communication across health sectors are fragmented, inconsistent, and often informal. Although some reporting structures exist, nearly half of the respondents work in areas without a clear chain of command for Mpox case reporting. The mixed ratings on communication effectiveness between federal and regional authorities reflect the absence of cohesive operational protocols. Furthermore, community health workers and NGOs, key actors in grassroots surveillance and response, are underutilized. The predominant use of mobile messaging platforms highlights both innovation and necessity in the absence of robust institutional systems. However, it also emphasizes the need for formalized, scalable communication structures that support timely information sharing and coordination during public health emergencies.

Preparedness across organizations is uneven and frequently inadequate. The data revealed that more than half of the organizations lack contingency plans, and very few conduct simulation exercises to test response capacity. The absence of PPE stockpiles, low levels of trained staff, and fragmented communication systems present serious barriers to an effective Mpox response. Although 45.3% of respondents viewed their organizations as capable or highly capable of managing an outbreak, confidence remained limited because over half perceived only partial or no readiness. These gaps are compounded by severe underfunding, 78.6% rated existing financial support as inadequate, further eroding the ability to develop, sustain, and operationalize essential preparedness functions. Based on the respondents’ reported preparedness gaps, simulation exercises, emergency drills, and sustainable funding mechanisms appear important for strengthening Mpox outbreak readiness.

Structural inequities also emerged as a defining theme in the assessment. Disparities in access to training, diagnostics, and public health education between urban and rural areas were widely reported. Surveillance systems often overlook marginalized populations, with over two-thirds of respondents noting the absence of gender-disaggregated data. The inconsistent use of local languages in community engagement further marginalizes underserved populations. Policy gaps and the lack of inclusive planning contribute to low levels of trust and engagement, particularly in communities most at risk. Respondents emphasized the need for localized outreach, linguistically and culturally tailored education, and the incorporation of community leaders and CHWs into national preparedness efforts. These findings suggest that equity-related considerations may play an important role in strengthening preparedness and public health response capacity.

The respondents’ perspectives highlight the potential value of a more integrated and inclusive Mpox preparedness strategy. Respondents strongly advocated for the integration of Mpox into national HMIS and PHEM systems, the decentralization of laboratory testing, and routine training for health professionals at all levels. They also called for stronger multisectoral coordination under a One Health framework, recognizing the zoonotic origins of Mpox and the interconnectedness of human, animal, and environmental health. Importantly, building trust and accountability through inclusive governance, equitable resource allocation, and community-driven engagement will be essential to addressing the preparedness gaps identified in this study and strengthening Ethiopia’s capacity for Mpox surveillance and response.

This study has several limitations. First, the reliance on self-reported survey data introduces potential social desirability and recall biases, as respondents may have over- or under-estimated their knowledge levels or the capacity of their institutions. Second, the cross-sectional design provides only a snapshot in time, limiting the ability to assess temporal trends or changes in preparedness. Third, although efforts were made to ensure a diverse sample, the respondents may not fully represent all regions or facility levels, particularly underrepresented rural settings. Fourth, only 9.5% of respondents were female, which may underrepresent women’s perspectives, especially on gender-sensitive surveillance and equity considerations. Finally, some qualitative responses were brief, restricting deeper exploration of complex systemic and contextual challenges. Nonresponse bias may have affected the findings, as not all eligible participants responded to the survey, potentially limiting representativeness.

Future research should focus on longitudinal assessments to track changes in Mpox preparedness and the effectiveness of interventions over time. Implementation research could help identify best practices for integrating Mpox into existing health systems, and operational studies could evaluate the scalability and impact of decentralized diagnostic platforms. Further investigation into community engagement strategies and culturally appropriate risk communication approaches will be essential for equitable health interventions. Finally, disaggregated studies exploring the experiences of women, marginalized populations, and frontline health workers could offer valuable insights into equity gaps and inform more inclusive policy development.

The study identified several priorities perceived as important for strengthening Mpox preparedness. These included the development of a national Mpox-specific preparedness and response plan, the integration of Mpox-specific modules into digital reporting platforms, institutionalized CHW engagement frameworks, and multilingual, culturally grounded public education strategies. Participants emphasized the need to move beyond general zoonotic preparedness toward more specific and actionable Mpox guidance, alongside broader engagement among public health stakeholders, NGOs, and communities around shared preparedness goals. Overall, the perspectives gathered in this study indicate that more coordinated, equity-centered, and technically grounded approaches may enhance preparedness for Mpox and other emerging public health threats.

### Recommendations

1. Establish standardized Mpox training pathways tailored to different roles in the health system.
2. Embed Mpox alerts and case definitions into DHIS2 and Kobo Toolbox platforms.
3. Launch policy-backed weekly surveillance meetings across all administrative levels.
4. Decentralize and institutionalize simulation exercises at the district level.
5. Map and monitor CHW engagement and formally include them in national surveillance.
6. Develop modular, locally translated Mpox risk communication kits.
7. Institute real-time feedback loops for reporting and outbreak information.
8. Create an Mpox Equity Checklist for facility-level assessments.
9. Introduce a national Mpox preparedness and response plan distinct from general zoonotic policies.
10. Mandate the inclusion of Mpox in IDSR reporting tools and health workforce key performance indicators (KPIs).
11. Engage universities and regional health bureaus in co-developing context-specific training materials.
12. Establish a public-facing Mpox preparedness dashboard with localized data, updated weekly.
13. Incorporate gender, age, and geographic equity indicators into national preparedness audits.
14. Fund civil society organizations to support outreach and misinformation countermeasures in remote areas.
15. Pilot One Health coordination frameworks at the regional level, with joint reporting and response protocols.
16. Design and roll out mobile-compatible Mpox surveillance and reporting apps for remote and offline use.
17. Invest in nationwide training for frontline staff on digital surveillance tools and data quality standards.
18. Create a centralized e-learning portal for Mpox outbreak management linked to MoH and EPHI systems.
19. Conduct digital infrastructure audits across health facilities to inform strategic IT investment.
20. Establish national digital health governance standards that integrate Mpox into broader eHealth architecture.

## Conclusion

Ethiopia’s Mpox preparedness, as reflected in the perceptions of the 42 surveyed surveillance professionals, illustrates how a combination of perceived system limitations, resource inequities, and underutilized technologies may influence vulnerability within surveillance practice. While respondents reported considerable awareness of Mpox and its potential public health threat, they also described challenges in translating this awareness into operational readiness within their respective settings. More than isolated deficiencies, the findings suggest the presence of perceived structural constraints characterized by a recurring mismatch between institutional intent and ground-level execution. The continued reliance on manual reporting systems, limited scope of training, insufficient diagnostic capacity, and weak integration of Mpox surveillance into digital platforms were viewed by respondents not only as technical gaps but also as possible reflections of broader challenges related to prioritization, resource allocation, and system coordination. In an era where digital transformation is increasingly central to health system resilience, these perceptions highlight the potential importance of implementation fidelity, cross-sector coordination, and inclusiveness in surveillance practice. Within the context of this study, preparedness for Mpox was not perceived as a standalone intervention but rather as an expression of how effectively health system components are adapted and operationalized in routine practice. Rather than serving as a national-level judgment, these findings should be understood as reflecting the perceptions of the surveyed professionals, offering contextual insights that may inform efforts to strengthen training, improve digital system integration, and enhance coordination within comparable surveillance settings in Ethiopia.

## Supplementary Information

Supplementary material 1.

Supplementary material 2.

Supplementary material 3.

## Data Availability

The datasets used and/or analyzed during the current study are provided as Supplementary File 3.

## Abbreviations

AI: Artificial Intelligence
Africa CDC: Africa Centres for Disease Control and Prevention
CBS: Community-Based Surveillance
CDC: Centers for Disease Control and Prevention
CHWs: Community Health Workers
CRISPR: Clustered Regularly Interspaced Short Palindromic Repeats
crRNA: CRISPR RNA
dPCR: Digital Polymerase Chain Reaction
DHIS2: District Health Information Software 2
DRC: Democratic Republic of the Congo
ELISA: Enzyme-Linked Immunosorbent Assay
EPHI: Ethiopian Public Health Institute
FCDO: Foreign, Commonwealth and Development Office
HCW: Healthcare worker
IHC: Immunohistochemistry
IDRC: International Development Research Centre
IDSR: Integrated Disease Surveillance and Response
KAP: Knowledge, Attitude, and Practice
LAMP: Loop-Mediated Isothermal Amplification
LFD: Lateral Flow Dipstick
mHealth: Mobile Health
mNGS: Metagenomic Next-Generation Sequencing
MoH: Ministry of Health
MPXV: Monkeypox Virus
NGS: Next-Generation Sequencing
NRL: National Reference Laboratory
NSERC: Natural Sciences and Engineering Research Council of Canada
OPXV: Orthopoxvirus
PCR: Polymerase Chain Reaction
PHEM: Public Health Emergency Management
PHEOC: Public Health Emergency Operations Center
POCT: Point-of-Care Testing
PPE: Personal protective equipment
qPCR: Quantitative polymerase chain reaction
RAA: Recombinant enzyme-assisted amplification
RCCE: Risk communication and community engagement
RDTs: Rapid diagnostic tests
RPA: Recombinant polymerase amplification
RRL: Regional Reference Laboratory
RT-PCR: Reverse Transcription Polymerase Chain Reaction
UN2814: United Nations Infectious Substance Classification Code 2814
WGS: Whole-Genome Sequencing
