# Supplementary material for: A rapid evaluation of the preparedness of Ethiopia's disease surveillance system for Mpox outbreak: a cross-sectional study of perspectives from professionals across various levels
Source: Trop Med Health. 2026 Jun 6;54:120. doi: 10.1186/s41182-026-00994-8 (PMC13285013; doi:10.1186/s41182-026-00994-8)
Supplement: Supplementary file 1 — Supplementary material 1. [file 41182_2026_994_MOESM1_ESM.pdf]

STROBE Checklist for rapid evaluation of the preparedness of Ethiopia's disease surveillance

Section	STROBE Item	Requirement	Status	Page/Section Reference
Title & Abstract	Study design	Mixed-methods cross-sectional design clearly stated	Completed	Title page; Abstract
	Abstract	Includes setting, participants, and key findings	Completed	Abstract
Introduction	Background	Surveillance gaps and language barriers are described	Completed	Introduction
	Objectives	Clear objectives on usability, integration, and multilingual performance	Completed	Introduction
Methods	Study design	Cross-sectional mixed-methods specified	Completed	Methods
	Setting	The Ethiopian surveillance system and study context are described	Completed	Methods
	Participants	Inclusion criteria and sampling explained	Completed	Methods
	Variables	Key domains defined (usability, integration, etc.)	Completed	Methods
	Data sources	Questionnaire and evaluation described	Completed	Methods
	Bias (Item 10)	Bias and quality control measures addressed	Completed	Methods – Bias & Quality Control; Discussion – Limitations
	Study size (Item 7)	Sample size justification provided	Completed	Methods – Study Design (exploratory study)
	Statistical methods (Item 12e)	Descriptive and thematic analysis are described, including handling of missing data	Completed	Methods – Data Analysis (data completeness check; listwise deletion/handling approach)
Results	Participants (Item 13a–16)	Sample size, distribution, descriptive data, and outcomes reported	Completed	Results

	Outcome data	Findings aligned to study domains	Completed	Results
	Main results	Results are data-driven	Completed	Results
	Flow diagram (Item 13c)	Flow diagram	Not Applicable	Not used/stated in checklist
Discussion	Key findings	Main findings summarized	Completed	Discussion
	Limitations (Item 19)	Study limitations and non-response bias are clearly stated	Completed	Discussion – Limitations
	Interpretation	Interpretation linked to findings	Completed	Discussion
	Generalizability	Applicability discussed	Completed	Discussion
Other	Funding (Item 22)	Funding sources reported	Completed	Funding/Acknowledgements
	Ethics	Ethical approval and consent were described	Completed	Ethics Statement
