# Supplementary material for: A rapid evaluation of the preparedness of Ethiopia's disease surveillance system for Mpox outbreak: a cross-sectional study of perspectives from professionals across various levels
Source: Trop Med Health. 2026 Jun 6;54:120. doi: 10.1186/s41182-026-00994-8 (PMC13285013; doi:10.1186/s41182-026-00994-8)
Supplement: Supplementary file 2 — Supplementary material 2. [file 41182_2026_994_MOESM2_ESM.pdf]

Questionnaire for evaluating the readiness and preparedness of the Ethiopian disease surveillance system for MPox

Notice to Respondents

Dear Respondents,

This questionnaire is designed to assess the readiness and preparedness of Ethiopia's public health surveillance system for the Mpox virus. The data collected will help identify strengths, gaps, and opportunities for improvement, guiding efforts to enhance surveillance and response capabilities. Your responses are confidential and will be used solely for research and policy development purposes.

The questionnaire has demographic information section and five parts. Please provide accurate information~~

* Indicates required question

1. Phone number

2. Email

Demographic Information

Please fill out the following details before proceeding with the questionnaire.

3. Full Name *

4. Organization *

5. Role in the organization *

6. Organization type *

Mark only one oval.

- Federal
- Regional
- Zonal
- Woreda
- Facility
- Other: ____________________

7. Years of experience in public health surveillance *

Mark only one oval.

- <1 year
- 1-5 years
- 6-10 years
- Option 4
- > 10 years

8. Primary area of responsibility *

Mark only one oval.

- Disease surveillance
- Outbreak response
- Data collection and analysis
- Community engagement
- Policy and planning

9. Gender *

Mark only one oval.

- Male
- Female
- Prefer not to say

Part 1: General Awareness and Understanding (10 questions)

The following questions help understand the awareness and understanding pertaining to Mpox

10. How familiar are you with Mpox and its symptoms? *

Mark only one oval.

- Very familiar
- Familiar
- Somehow familiar
- Not at all familiar

11. Do you believe Mpox poses a significant public health threat in Ethiopia? *

Mark only one oval.

- Yes
- No

12. Which of the following do you associate with Mpox transmission? *

Mark only one oval.

- Animal contact
- Human-to-human contact
- Contaminated objects
- Don't know

13. Briefly describe the main sources of information you rely on for updates about emerging diseases. *

14. Rate the level of preparedness of Ethiopia's health surveillance system for handling emerging infectious diseases. *

Mark only one oval.

- Excellent
- Good
- Average
- Poor

15. How often are you updated on changes in infectious disease policies? *

Mark only one oval.

- Weekly
- Monthly
- Quarterly
- Rarely

16. Have you participated in any training related to Mpox or similar diseases? *

Mark only one oval.

- Yes
- No

17. If yes, specify the nature of the training.

____

18. How well do you think existing health infrastructure supports the detection of Mpox cases? *

Mark only one oval.

- Strongly supports
- Fairly supports
- Somewhat supports
- Does not supports

19. Rank the following sources of disease surveillance data in terms of reliability. *

Mark only one oval.

- Fairly reports
- Community reports
- Laboratory data
- Media monitoring

20. What gaps, if any, do you see in Ethiopia's ability to detect and respond to Mpox? *

____

Part 2: Surveillance Infrastructure and Resource (10 questions)

The following part delves into infrastructure and resources for Mpox.

21. Does your organization have dedicated staff for infectious disease surveillance? *

Mark only one oval.

- Yes
- No

22. Rate the adequacy of human resources for Mpox surveillance in your organization. *

Mark only one oval.

- Adequate
- Acceptable
- Inadequate

23. Are there sufficient laboratory facilities available for Mpox diagnostics in Ethiopia? *

Mark only one oval.

- Yes
- No

24. How accessible are laboratory services for Mpox testing in rural areas? *

Mark only one oval.

- Easily accessible
- Fairly accessible
- Option 3
- Somewhat accessible
- Not accessible

25. Describe the availability of technological tools (e.g., mobile apps, software) for data collection and reporting. *

26. What percentage of healthcare facilities in your jurisdiction are equipped to report suspected Mpox cases? *

Mark only one oval.

- > 75%
- 50-75%
- 25-50%
- <25%

27. Which resources are most lacking for effective Mpox surveillance in your area? *

Mark only one oval.

- Staff
- Equipment
- Training
- Funding

28. Are reporting mechanisms (e.g., DHIS2) in place to handle Mpox case data? *

Mark only one oval.

- Yes
- No

29. How often are routine disease surveillance meetings conducted at your level? *

Mark only one oval.

- Weekly
- Biweekly
- Monthly
- Quarterly

30. What improvements do you recommend for strengthening Mpox diagnostic capacity? *

Part 3: Coordination and Communication (10 questions)

The following section will help understand the coordination and communication aspects related to Mpox.

31. How well do public health organizations coordinate surveillance activities? *

Mark only one oval.

- Very well
- Well
- Somewhat
- Not at all

32. Is there a clear chain of command for reporting Mpox cases? *

Mark only one oval.

- Yes
- No

33. How effective are communication channels between federal and regional health offices? *

Mark only one oval.

- Effective
- Moderately effective
- Somewhat effective
- Ineffective

34. Are community health workers actively involved in disease surveillance in your jurisdiction? *

Mark only one oval.

- Yes
- No

35. What communication tools are most commonly used to share information about outbreaks? *

Mark only one oval.

- Emails
- Mobile apps (Telegram, Whatsapp, Messenger, etc.)
- Phone calls
- Paper reports

36. Describe any challenges in sharing data with other stakeholders. *

____
____
____
____
____

37. How quickly do you typically receive updates about emerging diseases from the Ministry of Health? *

Mark only one oval.

- Immediately
- Within 24 hours
- Within 3 days
- More than 3 days

38. Have any public awareness campaigns about Mpox been conducted in your area? *

Mark only one oval.

- Yes
- No

39. Rate the level of engagement of non-governmental organizations in surveillance activities. *

Mark only one oval.

- ◯ Highly engaged
- ◯ Engaged
- ◯ Less engaged
- ◯ Not engaged

40. What strategies do you suggest for improving coordination and communication among stakeholders? *

____
____
____
____
____

Part 4: Preparedness and Response (10 questions)

This section includes questions that seek to understand the preparedness and response aspects to Mpox.

41. Are there contingency plans for Mpox outbreaks at your level of operation? *

Mark only one oval.

- ◯ Yes
- ◯ No

42. Rate your organization’s capacity to respond to a Mpox outbreak. *

Mark only one oval.

- Highly capable
- Capable
- Somewhat capable
- Not capable

43. How many staff in your organization have been trained in outbreak response protocols for Mpox? *

Mark only one oval.

- >20
- 10-20
- <10
- None

44. Are there stockpiles of personal protective equipment (PPE) available for Mpox response? *

Mark only one oval.

- Yes
- No

45. How frequently are simulation exercises conducted for outbreak preparedness? *

Mark only one oval.

- Annually
- Biannually
- Rarely
- Never

46. What barriers would hinder effective Mpox outbreak response in your area? *

47. Do you have access to resources for public education about Mpox? *

Mark only one oval.

- Yes
- No

48. Rate the adequacy of funding allocated for disease outbreak preparedness. *

Mark only one oval.

- Adequate
- Somewhat adequate
- Inadequate

49. Are isolation and treatment facilities prepared to manage potential Mpox cases? *

Mark only one oval.

- Yes
- No

50. What key steps should be prioritized to improve preparedness for Mpox outbreaks? *

Part 5: Policy, Training, and Equity (10 questions)

The following questions will help understand the policy, training, and equity state and suggest possible best practices.

51. Are there existing policies in place for managing zoonotic disease outbreaks? *

Mark only one oval.

- Yes
- No

52. How well do current policies address the unique challenges of Mpox? *

Mark only one oval.

- Very well
- Well
- Somewhat
- Not at all

53. What training opportunities are available for staff involved in Mpox surveillance? *

____
____
____
____
____

54. Have staff in your organization received training on gender-sensitive approaches in surveillance? *

Mark only one oval.

- Yes
- No

55. Do current surveillance systems account for gender-disaggregated data collection? *

Mark only one oval.

- Yes
- No

56. Rate the inclusivity of marginalized groups in disease surveillance activities. *

Mark only one oval.

- Inclusive
- Less inclusive
- Not inclusive

57. Are local languages used in community outreach and case reporting? *

Mark only one oval.

- Yes
- No

58. What steps are being taken to involve underserved communities in Mpox preparedness? *

59. How can policies be adjusted to better support Mpox surveillance? *

60. What additional resources are needed to ensure equity in Mpox preparedness efforts? *

Thank you very much!

Gelan Ayana Zewdie (Ph. D.)

This content is neither created nor endorsed by Google.

Google Forms
